# Performance, Mechanical Properties and Durability of a New Type of UHPC—Basalt Fiber Reinforced Reactive Powder Concrete: A Review

**DOI:** 10.3390/polym15143129

**Published:** 2023-07-23

**Authors:** Fangyuan Li, Tangzhen Lv, Sihang Wei

**Affiliations:** 1 Department of Bridge Engineering, Tongji University, Shanghai 200092, China; 2132379@tongji.edu.cn; 2 Jujie Technology Development Group Co., Ltd., Ningbo 315615, China; phillipwsh@163.com

**Keywords:** basalt fiber reinforced reactive powder concrete, work performance, mechanical properties, durability

## Abstract

The advent of reactive powder concrete (RPC) has brought about the era of ultra-high performance concrete (UHPC), and the incorporation of fiber has brought about more possibilities for its application. Basalt fiber reinforced reactive powder concrete (BFRPC), as the product of the combination of RPC and fiber, has become a new engineering material that has received much attention from scholars in recent years. Compared with traditional UHPC, BFRPC is superior in corrosion resistance, material compatibility, cost performance, environmental protection, and other aspects; therefore, it is destined to have a wide range of applications in the future. In this article, we extensively reviewed the literature on basalt fiber reinforced RPC in the past decade from the perspective of work performance, mechanical properties, and durability. Moreover, we summarized the research progress and achievements on BFRPCs in the following points: (1) The performance of BFRPCs is mainly influenced by three factors: the frictional resistance between fine aggregates, the consistency of the cement slurry, and the three-dimensional random interweaving of basalt fibers; (2) the mechanical properties of BFRPC are mainly influenced by curing conditions, the design of the RPC matrix proportional mix, and the addition of basalt fibers up to a threshold; (3) thanks in part to RPC’s density and the filling and bridging of fibers, BFRPC exhibits uniform and good performance in durability indicators. However, there are still some problems in the current development of BFRPC, such as inconsistent test conclusions among different scholars and a lack of scenarios in which to apply BFRPC. This paper also puts forward the prospect from the aspects of theoretical research and practical application, and provides a reference for subsequent related work.

## 1. Introduction

In the past few decades, with the continuous development of the economy and society, significant achievements have been made in all aspects of civil engineering. Ultra-high performance concrete (UHPC), one of the important material advancements in the field of civil engineering in recent years, has demonstrated excellent performance in various aspects, such as work performance, mechanical properties, and durability. Moreover, UHPC has broad prospects for scientific research and market applications [1]. The concept of UHPC materials was first proposed in the 1990s [2]. Later, with the emergence of reactive powder concrete (RPC) [3], the era of concrete ultra-high performance with RPC materials officially arrived. As research on UHPC materials has deepened, their applications have become more widespread in fields such as bridge engineering, building engineering, and protective engineering [4].

Most of the UHPC materials currently being widely used are prepared by adding fiber admixtures to an RPC matrix material, with the RPC mix ratio as the core [5]. Due to the compactness of RPC materials and the bridging function of fiber admixtures, UHPC materials perform better in strength, durability, and crack resistance than high-performance concrete and ordinary concrete (Table 1). Since the mechanical and durability properties of concrete are greatly affected by its internal pore structure, RPC materials have abandoned the coarse aggregates in traditional concrete preparation processes, replacing them with finer aggregates in richer gradations to improve the density of the internal structure; at the same time, the high-density particles and fiber accumulation significantly reduced the porosity, and the connectivity of the material matrix’s internal pore was reduced, thereby having a high resistance to gas and liquid permeation. These factors have created conditions for improving the material’s mechanical and durability properties [6].

In addition, the incorporation of fiber provides more ideas for the preparation of UHPC materials. Inspired by the practice of incorporating straw into the soil in the past to delay wall cracking, researchers proposed the idea of using fiber to improve the cracking resistance of concrete in the early twentieth century [8]. Thanks to the continuous deepening of research, fiber-reinforced concrete materials have come into being. A large number of tests demonstrate that fiber incorporation not only fully realizes the goal of improving the cracking resistance of concrete, but also has a positive effect on the concrete’s mechanical properties, electrical properties, and durability [9,10,11]. However, it is worth noting that different types of fiber admixtures have varying effects on the performance improvement of UHPC materials. Inorganic fibers are generally used to improve the material’s strength, while organic fibers are mainly aimed at improving the material’s toughness [12]. Therefore, the selection of fiber type is also an important aspect of UHPC material in practical applications. Currently, common fiber admixtures in UHPC materials include steel fibers (SF), carbon fibers (CF), basalt fibers (BF), etc. (Table 2), with steel fibers being the most common. By changing the pouring method or applying an electromagnetic field, the steel fiber can be distributed parallel to the direction of the principal stress after being added. The ASFRPC (aligned steel fiber–reinforced reactive powder concrete) obtained in this preparation mode has 2–2.5 times the bending capacity of ordinary SFRPC (steel fiber reactive powder concrete) under the same conditions, which effectively improves its utilization rate and material properties [13]. The incorporation of CF can make the mechanical properties of RPC at −25 °C still superior to that of plain RPC [14]. The above studies indicate the great research significance and possible applications of fiber-reinforced active powder concrete. However, on the one hand, the problem of rusting steel fibers when exposed to the surface of the structure needs to be addressed; on the other hand, the poor economy of carbon fibers makes it temporarily difficult to become an ideal substitute for steel fibers. In this context, basalt fibers have gradually attracted the attention of researchers.

Basalt is a dense or foam-like structure of rocks condensed by volcanic magma to the surface and belonging to a class of magmatic rocks (igneous rocks) that are widely distributed in nature. Basalt can be quickly drawn into fibers after melting at 1500 °C. As a kind of continuous inorganic nonmetallic fiber (Table 3), the production process of basalt fiber is environmentally friendly and economical, making it an ideal industrial raw material [16]. On the one hand, as a product of volcanic rock firing, basalt fiber has the advantages of high strength, good corrosion resistance, and easy material acquisition. On the other hand, the chemical nature of basalt fiber is silicate, which has good compatibility with concrete, providing a good prerequisite for its use as a fiber admixture. The addition of basalt fiber can not only effectively solve the problem of corrosion in the application of steel fiber and the economic problem in the application of carbon fiber, but also improve the mechanical and durability properties of the material [17].

Basalt fiber reinforced reactive powder concrete (BFRPC) is a type of ultra-high performance fiber-reinforced concrete material with an RPC matrix material ratio as the core and basalt fiber as the reinforcement material. BFRPC has superior performance in corrosion resistance, material compatibility, cost-effectiveness, and environmental friendliness, indicating that it will have a wide range of applications in the future.

Based on the research results of BFRPC in the past decade at home and abroad, this article analyzes and summarizes the performance, mechanical properties, and durability of BFRPC and its influencing factors and proposes prospects for the current problems in the development of BFRPC materials at the research and application levels.

## 2. Performance of Basalt Fiber Reinforced Polymer Concrete (BFRPC)

Good performance of concrete guarantees construction efficiency and quality, and the flowability of concrete is one of the important indicators for evaluating the performance of concrete. The materials used in preparing BFRPCs in the literature are usually quartz sand, quartz powder, cement, silica fume, water-reducing agent, and basalt fiber [19]. As BFRPC is a reinforcement formed by adding basalt fiber to an RPC matrix material that eliminates the coarse aggregate, its flowability is mainly affected by three factors: the frictional resistance between fine aggregates, the consistency of cement paste, and the three-dimensional interweaving of basalt fibers. Among them, the former two are mainly determined by the proportion and types of materials added to the RPC matrix, while the three-dimensional interweaving of basalt fibers is mainly related to the amount and surface treatment of basalt fibers in BFRPC materials.

### 2.1. RPC Matrix

Scholars have mainly studied the proportion of materials added to the BFRPC matrix, such as the water–cement ratio, aggregate ratio, and cementitious materials. Liu, et al. [20] used the BDD response surface method and statistical parameters to study the factors affecting the flowability of RPC. The experimental results demonstrated that there were interactive effects between the sand–cement ratio and the silica fume–cement ratio and between the water–cement ratio and the silica fume–cement ratio in the factors affecting the flowability of RPC. The degree of influence of each factor is as follows: Water–cement ratio > silica fume–cement ratio > sand–cement ratio > interaction between sand–cement ratio and silica fume–cement ratio > interaction between water–cement ratio and silica fume–cement ratio.

**Figure 1:**
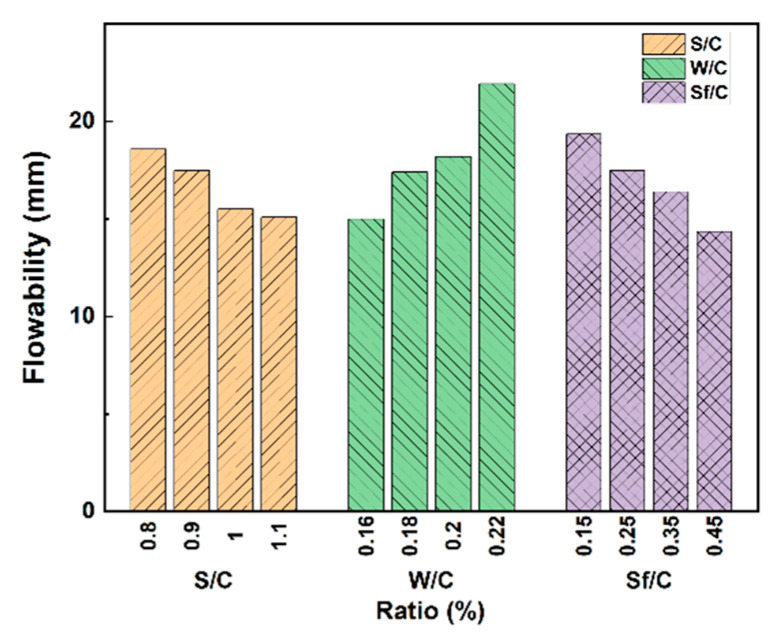
The influence of RPC matrix on BFRPC flowability [21]. S/C, sand–cement ratio; W/C, water–cement ratio; Sf/C, silica fume–cement ratio.

Figure 1 discusses the impact of sand–cement ratio, water–cement ratio, and silica fume–cement ratio on the flowability of BFRPC based on the results from a study [21]. Scholars have explored the substitution of different types of admixtures in the BFRPC matrix in pursuit of greater economic and environmental benefits. For example, He, et al. [22] conducted an orthogonal experiment on the design of BFRPC mix proportions by using fly ash to partially replace cement. Their experimental results demonstrated that although the addition of fly ash decreased the 28-day mechanical strength of BFRPC, when the replacement amount of fly ash to cement was 30%, the compressive strength of BFRPC only decreased by 6% compared to the control group, while the workability of RPC was significantly improved. This indicates that fly ash, which has a spherical particle shape, can play a good lubricating role in the cement slurry; therefore, the amount of fly ash substituting for cement will also be one of the important factors affecting the flowability of BFRPC. Meanwhile, Yao [23] designed and prepared a river sand-based BFRPC mix proportion, which substitutes natural river sand for quartz sand and mineral admixture for part of the cement, while ensuring the mechanical performance of the river sand-based BFRPC. The performance is also considered.

### 2.2. Basalt Fiber

**Figure 2:**
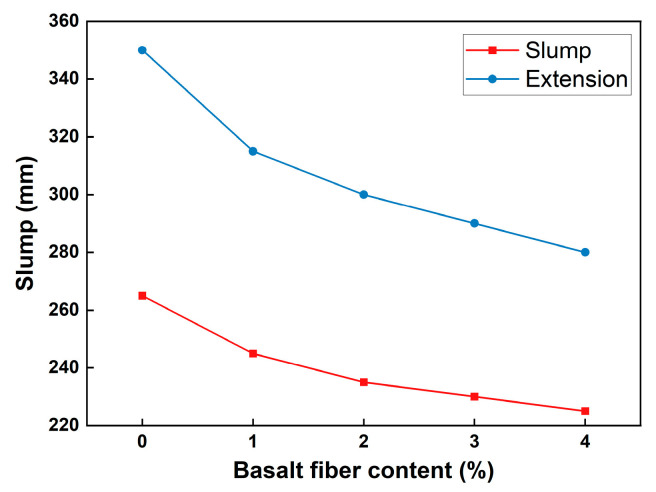
The influence of basalt fiber content on BFRPC flowability [25].

As an important component in the preparation of BFRPC, the inclusion of basalt fiber has brought new changes to the workability of the RPC matrix. Jia et al. [24] compared the differences in the influence on RPC matrix flowability between basalt fiber, steel fiber, and polypropylene fiber. The results demonstrated that although the inclusion of fiber reduces the flowability of the RPC matrix, due to the smaller diameter of basalt fiber relative to the other two fibers, the number of fibers per unit area under the same fiber content is far greater than the other two fibers, and its impact on the flowability of the RPC matrix is more significant. Liu et al. [25] conducted tests on the slump and spread of BFRPC, with fiber contents ranging from 0% to 4%. As the content of basalt fiber increased, the flowability of RPC gradually decreased, and the rate of decrease gradually slowed down (Figure 2).

The inclusion of basalt fibers will reduce the flowability of the BFRPC mixture [26]. On the one hand, because part of the cement slurry will adsorb and wrap around the basalt fibers, the amount of free-flowing cement slurry is reduced; on the other hand, the inclusion of basalt fibers forms an interlaced spatial network structure after mixing, which restricts the free flow of various components inside the RPC matrix. As the content of basalt fiber increases, the internal disordered and interlaced structure of its spatial network becomes more complex and dense, increasing the limiting effect and requiring more cement slurry to wrap around the basalt fiber, which in turn reduces the amount of free-flowing cement slurry and ultimately causes the flowability of the BFRPC mixture to continuously decrease.

The surface treatment of basalt fibers also affects the flowability of the BFRPC mixture. [27] explored the influence of different surface treatment methods on the flowability of the BFRPC mixture by treating basalt fibers soaked in water in different ways. The experimental results demonstrated that after basalt fibers were treated with the silane coupling agent KH-550, the flowability of the BFRPC mixture was generally improved, and the degree of improvement was almost linearly related to the mass fraction of the silane coupling agent. Compared with the untreated control group, its flowability was maximally increased by 22.8%. When basalt fibers treated with hydrochloric acid etching were included in the BFRPC mixture, the flowability of the BFRPC mixture exhibited a decreasing trend, and the concentration of hydrochloric acid, etching time, and etching temperature all affected this trend. Obviously, the two different surface treatment forms affect the contact between basalt fibers and the RPC matrix: the surface of the basalt fibers treated with the silane coupling agent formed a thick silane layer, reducing the friction between the basalt fibers and the RPC matrix and improving the flowability of the BFRPC mixture, while the surface of the basalt fibers treated with acid etching increased the roughness and the contact area between the basalt fibers and the RPC matrix.

### 2.3. Preparation Process

After determining the base material ratio of the BFRPC mixture to a certain extent, the specific preparation process needs to be discussed. Ensuring the uniform distribution of all components in the matrix is also an important aspect of concrete’s performance. Compared with steel fiber, basalt fiber is more flexible, and it is usually coated with plastic before packaging to prevent surface oxidation. Therefore, the preparation process of BFRPC is different from that of traditional UHPC, which is mainly composed of SF. [28] proposed two preparation methods according to the different incorporation methods of basalt fiber:

1. Direct mixing method: The basalt fiber is first separated, and then the basalt fiber is directly mixed into BFRPC during mixing according to the way of steel fiber.
2. Pre-saturation method: The basalt fiber is first soaked with tap water to saturate it, and then it is mixed into RPC mortar to form BFRPC.

**Figure 3:**
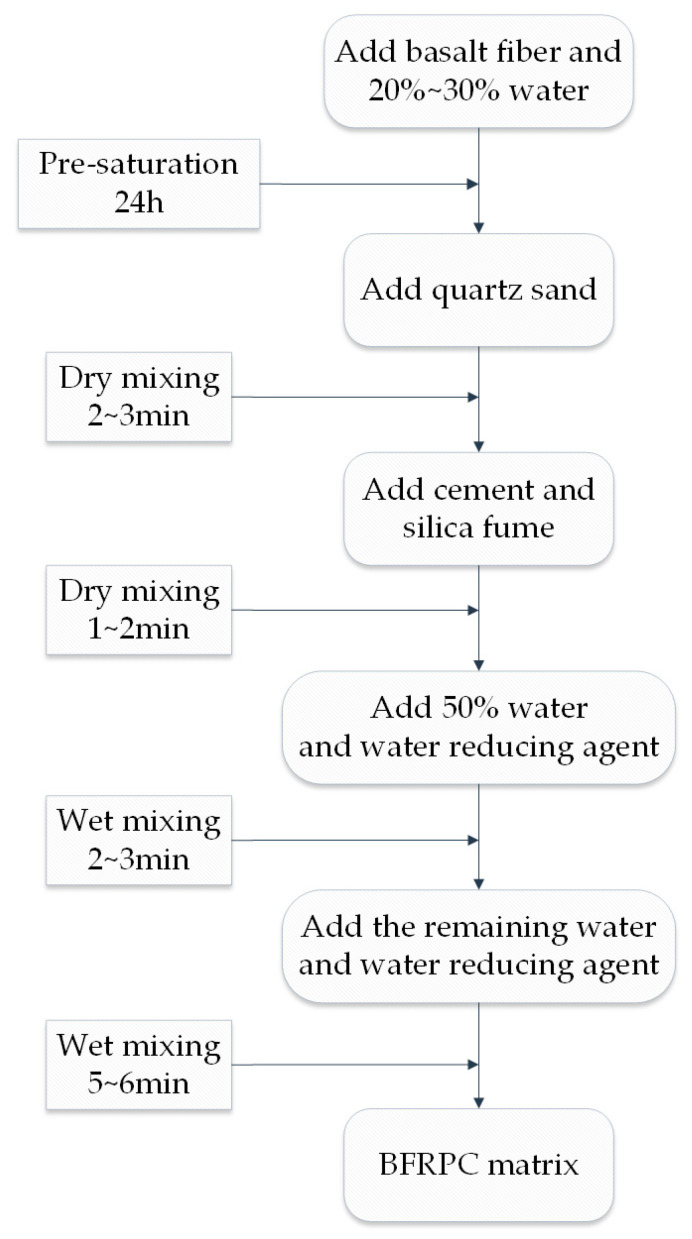
BFRPC matrix preparation process.

The pre-separation in the direct mixing method is to avoid as much as possible the problem of electrostatic adsorption caused by the fiber indication gum. However, this method is more complicated and inconvenient to operate. Therefore, the convenient and simple pre-saturation method is recommended to prepare BFRPC. The pre-saturation method can not only wash away the surface glue to a certain extent, but also solve the electrostatic attraction by taking advantage of the large water absorption of the basalt fiber, so that the components of the BFRPC matrix are more evenly distributed in the agitation. Figure 3 shows the preparation process of the BFRPC matrix.

## 3. Mechanical Properties of Basalt Fiber Reinforced Active Powder Concrete (BFRPC)

The mechanical properties of active powder concrete with basalt fiber (BFRPC) are a key index to evaluate structural safety. As a new type of concrete material, the mechanical properties of BFRPCs have been the focus of scholars’ attention. At present, extensive research has been carried out on the factors that affect the mechanical properties of BFRPCs, such as curing conditions, RPC matrix mix design, and the inclusion of basalt fibers.

### 3.1. Curing Conditions

**Figure 4:**
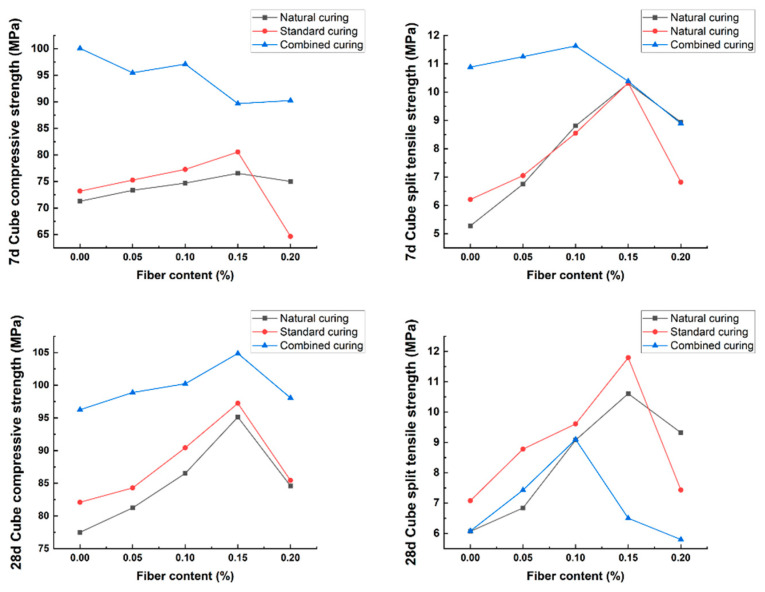
Compressive and splitting strength of BFRPC under different curing regimes [30].

Curing conditions affect the cement hardening rate and microporous structure of BFRPC, thereby affecting its mechanical properties. Shan [29] studied the effect of different curing regimes on the compressive strength of BFRPC and found that under high humidity and temperature curing conditions, the compressive strength of BFRPC at 28 d is higher. Moreover, the improvement effect of different curing conditions is as follows: Hot water curing combined with dry heat curing > hot water curing combined with standard curing > hot water curing > standard curing, with a maximum improvement of 121.38% between them. Zhou [21] analyzed the effect of different water bath curing temperatures (50 °C, 70 °C, and 90 °C) on the flexural and compressive strength of BFRPC and concluded that compared with standard curing, the flexural strength of BFRPC under high-temperature water bath curing was significantly improved in the early and late stages, with a maximum improvement of 41.78% at 28 d. The compressive strength of the BFRPC under high-temperature water bath curing developed rapidly in the early stage, and the strength at 3 d was the highest, with an increase of nearly 75% compared with the standard curing condition, approaching the strength at 28 d. However, with increasing curing age, the growth rate of the compressive strength gradually decreased. According to the test results of the compressive and splitting strengths of BFRPC under different curing conditions of natural curing, standard curing, and combination curing by Zhang et al. [30], the early compressive and splitting strengths of BFRPC under combination curing were the best. However, it should be noted that with increasing curing age, the splitting strength of BFRPC under combination curing did not increase significantly and even appeared to be “shrinking”. For the maximum improvement in compressive and splitting strength, the recommended fiber content of BFRPC was 0.15% for natural curing and standard curing and 0.10% for combination curing (Figure 4).

Combining the above research, it can be seen that under high-temperature and high-humidity curing conditions, the hydration reaction of BFRPC inside progresses more rapidly and sufficiently, and the early mechanical properties of BFRPC are greatly improved. However, under high-temperature and dry heat curing, the late-term mechanical property growth of BFRPC is slow, and there may even be a phenomenon of “shrinkage”. This is also related to the rapid progress of the cement hydration reaction. In the cement hydration reaction, hydration products are produced in large quantities in a short period and wrapped on the surface of cement particles, which reduces the efficiency of the hydration reaction, and the remaining amount of reactants available for the hydration reaction is small because most reactants are consumed in a short time.

### 3.2. RPC Matrix

**Figure 5:**
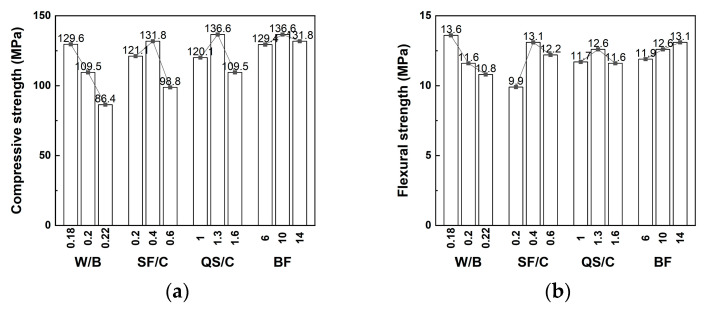
The influence of RPC matrix on (**a**) compressive strength and (**b**) flexural strength of BFRPC [31]. W/B, water/binder; SF/C, silica fume/cement; QS/C, quartz sand/cement; BF, basalt fiber.

The mechanical properties of BFRPC mainly depend on the RPC matrix. Liu et al. [31] designed the mixture ratio and strength test of BFRPC. The results demonstrated that the compressive and flexural strength of BFRPC decreased as the water–cement ratio increased, but that they increased first and then decreased as the silica–cement and sand–cement ratios increased (Figure 5). This is because, on the one hand, as the water–cement ratio increases, the free water content in the BFRPC mixture increases continuously, and the friction between the aggregates and the bonding force between the cementitious materials and the aggregates decrease, resulting in the continuous decline of the mechanical properties of BFRPC; on the other hand, as the sand–cement ratio increases, the mechanical strength of BFRPC shifts from the support effect of the fine aggregate skeleton to the bonding force of the cementitious materials, resulting in the increase and then decrease in the mechanical strength of BFRPC.

### 3.3. Basalt Fiber

The influence of basalt fiber mixing on the mechanical properties of BFRPC is of great significance. The fiber content and length are the research focus of scholars. The addition of basalt fiber changes the failure mode of RPC. Compared with plain RPC, which only exhibits tensile failure in the uniaxial compression process, the failure mode of BFRPC gradually transitions from tensile shear failure to shear failure with increasing basalt fiber content [32,33]. Table 4 summarizes the mechanical properties of basalt fiber RPC in recent years.

**Figure 6:**
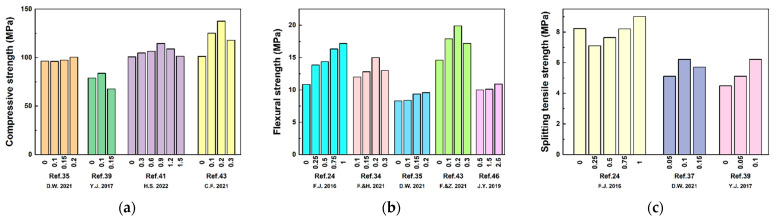
The results of BFRPC with the basalt fiber volume fraction. [24,34,35,37,39,41,43,46] (**a**) Compressive strength; (**b**) flexural strength; (**c**) splitting tensile strength.

In addition, many studies [42,43,44,45,46] have investigated the effects of basalt fiber reinforcement on the mechanical properties of BFRPC from various aspects, such as fiber content and length. Figure 6 shows the study results of the effect of basalt fiber content on the mechanical properties of BFRPC.

**Figure 7:**
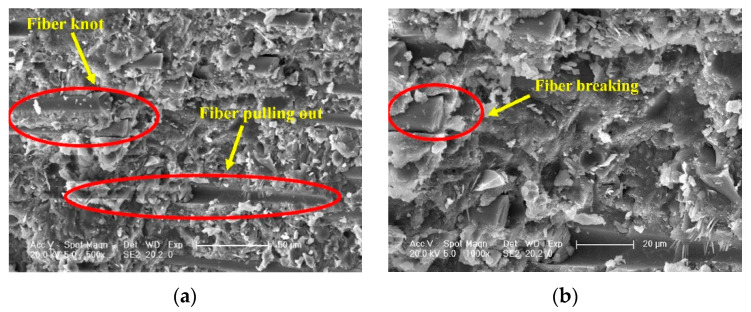
BFRPC microstructure [25]. (**a**) Fiber overlap and pulling out. (**b**) Fiber break.

Based on the fiber spacing theory [47] and microscopic results obtained by [25] in SEM characterization analysis (Figure 7), the reasons for the effect of basalt fiber reinforcement on RPC mechanical properties are analyzed: Basalt fibers typically have much higher tensile strength than cement-based materials, and they are compatible with cement-based materials, which enables them to be uniformly distributed in the RPC matrix after mixing. The bond between the two is tight, there is no interfacial transition zone, and the fiber bridging effect is better; moreover, the connective matrix formed after combination can improve the weak defect areas that originally exist in the RPC matrix, optimize the structure of internal pores, and ultimately enhance the bending and tensile strength of the unreinforced RPC. At this point, the higher the basalt fiber content and the longer the fiber length in the unit BFRPC volume, the more significant the enhancement effect will be. Therefore, the mechanical strength of BFRPC is positively correlated with fiber reinforcement. However, when the basalt fiber content and length exceed a certain range, fiber clustering and overlapping phenomena become obvious. At this time, the fiber is difficult to distribute uniformly, and it is difficult for it to be enveloped by the cement slurry, which creates a large number of new defect areas in the matrix, exceeding the strengthening effect of the basalt fiber on the RPC matrix strength, resulting in a decrease rather than an increase in the mechanical strength of the BFRPC.

It is worth mentioning that almost all of the abovementioned literature is about the static mechanical properties of BFRPC. There is relatively little exploration of the dynamic mechanical properties of BFRPC at present. [48] evaluated the dynamic response and damage of BFRPC bidirectional slabs under explosive loads with different fiber contents. With an increase in fiber content, the pressure and impulse required for the BFRPC bidirectional slab to reach the same damage level exhibited a tendency to first increase and then decrease. [49] conducted a high-speed impact test on BFRPC with different fiber volume contents. the results of the test demonstrated that the dynamic compressive strength of BFRPC was higher than its static compressive strength, but both exhibited a tendency to first increase and then decrease with an increase in fiber content. The strain rate effect of the BFRPC was significant, and its dynamic compressive strength, ultimate strain, impact toughness, and degree of fracture all increased with an increasing loading strain rate. Although BFRPC is a strain rate sensitive material, its strain rate sensitivity mainly comes from RPC itself, and the effect of basalt fiber reinforcement on strain rate sensitivity is small [50]. The bridging effect of basalt fiber reinforcement can suppress the development of microcracks within the concrete under dynamic impact caused by energy accumulation from loading, thereby effectively improving its dynamic mechanical properties [51].

## 4. Durability of Basalt Fiber Reinforced Powder Concrete (BFRPC)

Concrete is typically exposed to various environmental factors, such as rain, snow, and acid–base erosion, when used in practical applications. Therefore, the durability performance of materials plays a critical role in determining the service life and safety of structures in practice. The study of the durability performance of BFRPC materials is of great theoretical significance and engineering value. Scholars have mainly explored BFRPC in terms of its resistance to permeability, frost, and high temperatures.

### 4.1. Resistance to Permeability

Good resistance to permeability implies that the material can prevent adverse environmental factors from entering the structure, which reduces the safety of the structure. [52] analyzed the impact of the aspect ratio of basalt fibers on the BFRPC’s resistance to permeability and concluded that when the aspect ratio of basalt fibers was 6 mm/13 μm, BFRPC’s resistance to permeability was better than when the aspect ratio was 12 mm/15 μm. The resistance to permeability increased by up to 31.9% when the fiber content was 4 kg/m^3^. [53] used the electric flux method to study BFRPC’s resistance to permeability with different contents of basalt fibers. The experimental results demonstrated that with increasing fiber volume content, BFRPC’s resistance to permeability first increased and then decreased but was still better than plain RPC’s resistance to permeability. The electrical flux was less than 100 C, and the permeation of chloride ions could be ignored (Table 5). The current value of the BFRPC was significantly lower than that of the plain RPC, and its maximum decrease was 55.2%. Moreover, the growth of the current value was more gradual as the experiment progressed. BFRPC material has a dense RPC matrix and good resistance to permeability. After the addition of basalt fibers, according to the previous Section 1 the internal structure is optimized and the porosity is low, so its resistance to permeability is better than that of plain RPC [54,55].

### 4.2. Frost Resistance

To understand how to prevent concrete structures from being damaged in cold regions, the frost resistance of concrete materials has received wide attention from scholars. In the quantitative analysis of BFRPC durability by [57], it was found that crack width and freeze–thaw cycles have a significant impact on the durability of BFRPC. When the number of freeze–thaw cycles was 600, the quality loss rate of BFRPC subjected to the coupling effect of cracks and freeze–thaw cycles was 2.52%, which was higher than that without cracks and increased by 2.27%. However, it still met the requirement of a quality loss rate of no more than 5% in the specification. [36] tested the frost durability of different types of RPC fibers and found that the addition of basalt fibers can significantly improve the frost durability of the RPC matrix, and the frost durability of BFRPC increases with increasing fiber content. The damage to RPC under chloride salt freeze–thaw cycles is more severe than that under freshwater freeze–thaw cycles. Under the same fiber content, the addition of basalt fibers has a better effect on the frost durability of RPC than steel fibers. In addition, although the effect of carbonation on the mechanical strength of BFRPC is small [58], the coupling effect of chloride salt freeze–thaw cycles and carbonation can accelerate the attenuation of the mechanical strength of BFRPC. With increasing basalt content, the mechanical strength loss of BFRPC exhibits a quadratic decreasing function [59]. The bridging effect of basalt fibers inhibits the development of cracks, which is the main reason that BFRPC has better frost resistance than plain RPC.

### 4.3. High Temperature Resistance

The performance of concrete deteriorates significantly after being subjected to high temperatures. Therefore, observing the high-temperature resistance of BFRPCs is also an important issue in studying their durability. [60] studied the changes in the compressive strength of BFRPC at high temperatures with different fiber contents. The results demonstrated that with the increase in fire temperature, the mass loss rates of BFRPC under various fiber contents increased linearly, and their compressive strength first increased and then decreased, with mass loss rates lower than those of ordinary RPC. Although the bridging effect of basalt fiber can improve the mechanical properties of RPC to some extent after being subjected to high temperatures, its ability to inhibit high-temperature damage is limited [61]. Polypropylene fibers can form interconnected channels inside concrete after melting at high temperatures, ease the steam pressure inside concrete, and inhibit high-temperature cracking of concrete [62]. When mixed with basalt fibers and added to RPC, it can improve the high-temperature mechanical properties of RPC [63], which is a good idea to improve the mechanical properties of concrete after being subjected to high temperatures.

## 5. Summary and Outlook

### 5.1. Summary

Based on the literature in recent years, this article summarizes the various properties of BFRPCs and their influencing factors as follows:

- The performance of BFRPCs is mainly influenced by three factors: the frictional resistance between fine aggregates, the consistency of the cement slurry, and the three-dimensional random interweaving of basalt fibers. The former two are mainly determined by the proportion and type of admixture in the RPC matrix, while the three-dimensional interweaving of basalt fibers is mainly related to the amount and surface treatment of basalt fibers in BFRPC materials.
- The mechanical properties of BFRPC are mainly influenced by curing conditions, the design of the RPC matrix proportional mix, and the addition of basalt fibers. The mechanical strength of BFRPC mainly depends on the RPC matrix, while the curing conditions mainly affect the development of BFRPC strength. The strength of the BFRPC after being cured in hot water for 3 days reached 75% of the strength at 28 days.
- The addition of basalt fibers does not have a significant impact on the compressive strength of the RPC matrix but does significantly improve its bending and tensile strength. The improvement increases with the increase in the amount added, followed by a decrease.
- Thanks in part to the RPC’s own density and fiber-filling effect, as well as the bridging effect of basalt fibers, BFRPC exhibits uniform and good performance in durability indicators such as permeability resistance, carbonization resistance, frost resistance, and high-temperature resistance.

### 5.2. Outlook

Although the current research on BFRPC’s material properties has begun to take shape, based on the literature, scholars still have different conclusions on the relationship between the same influencing factors and related properties of BFRPC. In addition, BFRPC has been used less in practical engineering. To conduct more in-depth research on BFRPC materials and realize their engineering value, this article proposes the following considerations from the research and application levels:

- Different scholars have come to opposite conclusions regarding the relationship between the fiber length and the mechanical properties of BFRPCs. Obviously, the priority of the effect of fiber growth on the bridging ability and the strength attenuation caused by fiber aggregation will change with changes in the fiber length–diameter ratio and fiber content. Therefore, further research is needed on the complex relationship between the fiber length–diameter ratio and fiber content and the mechanical properties of BFRPCs.
- Currently, basalt fibers are usually mixed with other fibers for research on the dynamic mechanical properties of fiber-reinforced RPC. There is less research on the dynamic mechanical properties of BFRPC with single basalt fiber addition. Therefore, research should be conducted on the impact resistance and impact energy absorption capacity of BFRPCs.
- Steel fibers have an excellent load transfer capacity, and polypropylene fibers exhibit excellent high-temperature resistance. Combined with the advantages of basalt fibers, subsequent studies can try to conduct composite mixing of different types of fibers to configure fiber RPC materials that are more targeted and practical for engineering.
- SFRPC and CFRPC have been widely used in structural reinforcement, similar to the external steel plate method in strengthening methods, and demonstrate good increases in the bending and shear resistance of the structure. BFRPC exhibits better durability performance than SFRPC on the basis of meeting the mechanical properties. Moreover, BFRPC exhibits better economic performance than CFRPC. Therefore, BFRPC can be applied to structural reinforcement in a similar form.
- RPC components have the advantages of small size and light weight compared to ordinary concrete components, and they have good construction properties. In the future, BFRPC materials can be considered for use in the “lightweight” process of quality and cost in 3D printing of structures and preparation of decorative structural components.

## Figures and Tables

**Table 1:** Comparison of the main mechanical properties and durability of RPC, high-performance concrete (HPC), and ordinary concrete (OC) [7].

Concrete Type	Compressive Strength (MPa)	Flexural Strength (MPa)	Elastic Modulus (GPa)	Fracture Energy (kJ·m^2^)	Chloride Ion Diffusion Coefficient (m^2^/s)	Carbonization Depth (mm)	Freeze-Thaw Peeling (g/cm^2^)	Wear Coefficient
RPC800	409~705	45~140	63~74	1.2~2.0	-	-	-	-
RPC200	170~230	30~60	50~62	15~40	0.02 × 10^−12^	0	7	1.3
HPC	60~100	6~10	30~40	0.14	0.6 × 10^−12^	2	900	2.8
OC	20~50	2~5	30~40	0.12	1.1 × 10^−12^	10	>1000	4.0

**Table 2:** Comparison of the performance of different types of fibers [15].

Fiber Type	Density (g·cm^3^)	Tensile Strength (MPa)	Elastic Modulus (GPa)	Linear Expansion Rate (10^−6^·K^−1^)	Fracture Elongation (%)	Softening Point (°C)	Fabrication Temperature (°C)	Maximum Use Temperature (°C)
BF	2.56~3.05	3000~4840	79.3~93.1	6.5~8.0	3.1~3.2	960	1300	650
SF	7.8	380~1300	200	-	3~30	-	-	-
CF	1.78	2500~3500	230~240	0	1.2	-	-	-

**Table 3:** Main chemical composition of basalt (mass fraction, %) [18].

SiO_2_	Al_2_O_3_	Fe_2_O_3_ + FeO	CaO + MgO	Na_2_O + K_2_O	TiO_2_	Others
45~53%	12~16%	6~18%	10~20%	2~8%	1~5%	-

**Table 4:** Some research results on the effect of basalt fiber incorporation on the mechanical properties of RPC matrix.

Ref.	Inflecting Factor	Research Results
[34]	Fiber content	The addition of basalt fiber can improve the mechanical properties of RPC, but the improvement effect on different mechanical strength indicators is not the same. The improvement in flexural strength is more obvious than that in compressive strength.
[35]	Fiber content, fiber length	Based on SPSS analysis, the correlation coefficients between the addition of basalt fiber and the compressive strength, split tensile strength, and flexural strength of BFRPC are 0.737, 0.979, and 0.895, respectively.
[36]	Fiber content, fiber length	The addition of basalt fiber can improve the compressive and flexural strength of RPC. As the content of basalt fiber increases within 8–12 kg/m^3^, the compressive and flexural strength of BFRPC increases, with the maximum compressive and flexural strengths reaching 149.40 MPa and 16.23 MPa, respectively, which are 6% and 18.5% higher than those of plain RPC.
[37]	Fiber content, fiber length	The addition of basalt fiber has little impact on the early tensile strength development of BFRPC, but the maximum splitting tensile strength of BFRPC at 28 d is more than 30% higher than that of plain RPC. The splitting tensile strength of BFRPC is close to 1.382–2.263 times that of the axial tensile strength, and both increase and then decrease with the increase in basalt fiber content.
[38]	Fiber content	When the w/c ratio was maintained at 0.24, the compressive strength of BFRPC decreased with the increase in fiber content, and the compressive strength of BFRPC at 10 kg/m^3^ decreased by 18.2%, 7.8%, and 13.6% compared with plain RPC at 2, 7, and 28 days, respectively. With the increase in fiber content, the bending strength of RPC firstly increases (the maximum increase is 15.9%) and then decreases (the maximum increase is 17.7%). There is a linear relationship between wear resistance and compressive strength of BFRPC, and the coefficient of determination is 0.97.
[39]	Fiber content, fiber length	When the fiber length of 12 mm BFRPC is 0.10% by volume, the compressive strength and splitting tensile strength reach the maximum, which are 83.74 MPa and 6.22 MPa, respectively. When the volume content of BFRPC with a fiber length of 6 mm is 0.05%, the compressive strength and splitting tensile strength reach their maximums, which are 76.11 MPa and 6.05 MPa, respectively. Under the same fiber content, the BFRPC with a 6-mm fiber length will reach the maximum mechanical strength before that with a 12-mm fiber length.
[40]	Fiber length	When the basalt fiber length is 18 mm, the compressive strength of BFRPC continuously decreases with the increase in fiber content, but the compressive strength, flexural strength, and tensile strength of BFRPC at a 6-mm and 12-mm fiber length first increase and then decrease with the increase in fiber content. The bending and tensile strength of BFRPC with fiber volume content exhibited similar trends under different fiber lengths, but the fiber volume content was different when reaching the maximum strength, and the sequence was 18 mm before 12 mm before 6 mm.
[41]	Fiber content, fiber length	Through multiple linear fitting, the formula for calculating the relationship between fiber volume content, fiber length, and the compressive strength of BFRPC cube and prismatic body is proposed as follows: f_c_ = 1.046f_cu_ − 0.06L + 2.12v − 21.06 (R^2^ = 0.96896) The complete stress-strain curve prediction model of BFRPC was established.

**Table 5:** Concrete Chloride Ion Permeability Grade [56].

Charge Passed (Coulombs)	>4000	2000–4000	1000–2000	100–1000	<100
Chloride Ion Permeability	High	Moderate	Low	Very low	Negligible

## Data Availability

Not applicable.
